# Antioxidant properties of HDL

**DOI:** 10.3389/fphar.2015.00222

**Published:** 2015-10-16

**Authors:** Handrean Soran, Jonathan D. Schofield, Paul N. Durrington

**Affiliations:** ^1^Cardiovascular Research Group, Core Technology Facility, University of ManchesterManchester, UK; ^2^Cardiovascular Trials Unit, Central Manchester University Hospitals NHS Foundation TrustManchester, UK

**Keywords:** apolipoprotein A1, glycated low-density lipoprotein, high-density lipoprotein, oxidized low-density lipoprotein, paraoxonase 1

## Abstract

High-density lipoprotein (HDL) provides a pathway for the passage of lipid peroxides and lysophospholipids to the liver via hepatic scavenger receptors. Perhaps more importantly, HDL actually metabolizes lipid hydroperoxides preventing their accumulation on low-density lipoprotein (LDL), thus impeding its atherogenic structural modification. A number of candidates have been suggested to be responsible for HDL's antioxidant function, with paraoxonase-1 (PON1) perhaps the most prominent. Here we review the evidence for HDL anti-oxidative function and the potential contributions of apolipoproteins, lipid transfer proteins, paraoxonases and other enzymes associated with HDL.

## Introduction

Whilst atherogenesis is a complex process, macrophage-derived foam cell formation resulting from the uptake of circulating low-density lipoprotein (LDL) is of fundamental importance. Despite this, foam cell formation with LDL is impossible to instigate *in vitro* due to insufficient monocyte-macrophage LDL receptor expression (Steinberg and Witztum, 2010). It was, however, discovered that experimental chemical modification of LDL permitted its rapid receptor-mediated uptake by monocyte-macrophages to form foam cells. This led to the identification of the scavenger receptors (Stocker and Keaney, 2004; Steinberg and Witztum, 2010) and opened new avenues of research to identify possible *in vivo* atherogenic modifications of LDL. Oxidation, glycation, and homocysteinylation have all been explored. Although, clinical trials of chain-breaking antioxidants proved disappointing in the prevention of atherosclerotic cardiovascular disease (Heart Protection Study Collaborative, 2002), other systems which might oppose potentially atherogenic LDL modifications, including high-density lipoprotein (HDL) merit further attention.

We have contributed to the notion that paraoxonase 1 (PON1), an enzyme located almost exclusively on HDL, is important in impeding oxidative modification of LDL (Mackness et al., 1991, 1993; Durrington et al., 2001). Our recent work has focused on glycation as an atherogenic modification of LDL and this too has led us back to PON1 (Younis et al., 2013). Other HDL components have also been conjectured to be involved in preventing atherogenic LDL modification and evidence increasingly points to a coordination of these with PON1. Understanding these protective mechanisms might reveal important pathways which could be manipulated therapeutically to prevent atherosclerosis.

## Atherogenic LDL modification

The discovery that chemical modification of LDL by acetylation increases its affinity for macrophage scavenger receptors and reduces binding to the physiological LDL receptor led to a search for naturally occurring modifications which might have similar effects (Steinberg and Witztum, 2010). The ensuing hypothesis that lipid peroxidation products produced on LDL when subjected to attack by oxygen free radicals are responsible for changes in the apolipoprotein B100 (apoB) of LDL which alter its receptor binding preferences remains compelling despite the disappointing lack of effect of chain-breaking antioxidants (Heart Protection Study Collaborative, 2002; Stocker and Keaney, 2004; Steinberg and Witztum, 2010). Perhaps unsurprisingly given the safeguards against oxygen free radical damage, oxidized LDL is only found at low circulating concentrations, although it has been argued that higher levels might occur at sites where LDL is sequestered and this might include the arterial wall (Stocker and Keaney, 2004).

We were surprised to discover that glycated apoB was present in the circulation at relatively high concentrations of around 2–3 mg/dl in healthy people and at higher levels in diabetes and in hypercholesterolaemia (Tames et al., 1992). This has been confirmed by immunoassays detecting epitopes unique to glycated LDL and we have also shown that atherogenic small dense LDL is more heavily glycated than other LDL subfractions *in vivo*, and is more susceptible to glycation *in vitro* (Younis et al., 2009, 2010). Glycated LDL also has a longer circulating half-life than unmodified LDL, and is removed from the circulation by route(s) not involving the LDL receptor. Interestingly, statins also reduce circulating concentrations, likely by reducing LDL available to undergo glycation (Younis et al., 2010).

Homocysteine may also be atherogenic; thiolation of LDL free amino groups by homocysteine thiolactone increases its uptake by macrophages (McCully, 1993). Interestingly the lactonase activity of PON1 will detoxify homocysteine thiolactone in addition to its role in preventing LDL oxidation (see later).

## HDL antioxidative activity

**Figure 1:**
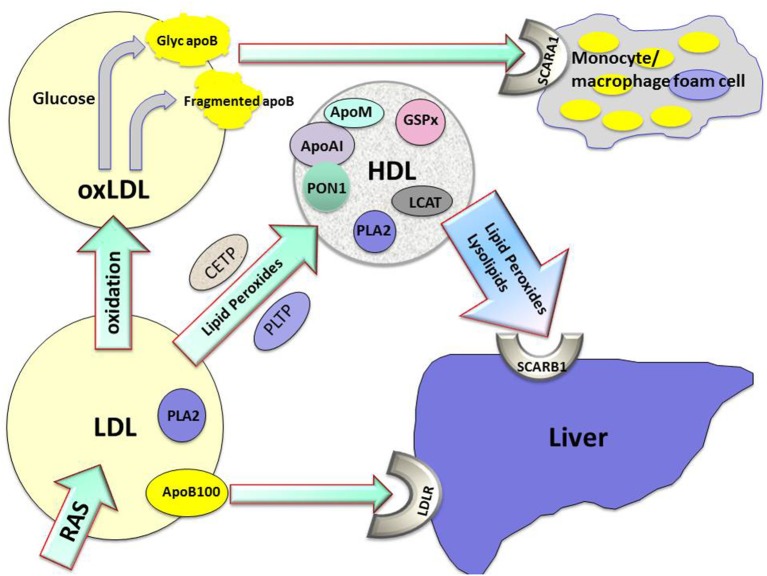
**The role played by high density lipoprotein (HDL) in the metabolism of lipid hydroperoxides and lysolipids and protection against atherogenesis**. Apo AI, apolipoprotein AI; apoB100, apolipoprotein B100; apoM, apolipoprotein M; CETP, cholesteryl ester transfer protein; glyc apoB, glycated apolipoprotein B; GSPx, glutathione peroxidase; LDL, low density lipoprotein; LDLR, low density lipoprotein receptor; oxLDL, oxidized low density lipoprotein; PLA2, phospholipase A2; PLTP, phospholipid transfer protein; PON1, paraoxonase1; RAS, reactive oxygen species; SCARA1, scavenger receptor A1; SCARB1, scavenger receptor B1.

Lipid hydroperoxides formed on LDL will migrate to its surface as a result of their greater hydrophilicity, facilitating their transfer to HDL (Parthasarathy et al., 1990). This transfer can occur directly between lipoprotein phospholipid monolayers, but may be assisted by lipid transfer proteins (see Figure 1). HDL might thus provide a pathway for the passage of lipid peroxides and lysophospholipids to the liver via hepatic scavenger receptors. Perhaps more importantly, HDL actually metabolizes lipid hydroperoxides preventing their accumulation, consequently impeding the atherogenic structural modification of LDL (Mackness et al., 1991). We have observed that when HDL is incubated with LDL under oxidizing conditions the accumulation of lipid peroxides on LDL is decreased, but the concentration of lipid peroxides on HDL remains similar to that observed when HDL alone is oxidized (Mackness et al., 1991, 1993). This effect of HDL is obvious within 3 h, by which time typically more than 50% of the lipid peroxidation of LDL which would occur in the absence of HDL has been prevented. These results suggest that this effect is related to enzymatic activity associated with HDL, and not chain-breaking antioxidants or transition metal chelation (Mackness et al., 1993; Durrington et al., 2001). It should also be noted that this anti-oxidative function of HDL is observed *in vitro* with similar protein concentrations of LDL and HDL; greater suppression of LDL oxidation might be expected when HDL concentrations exceed those of LDL as they do in the interstitial fluid. In fact, the accumulation of oxidized lipids in HDL likely results not only from their transfer from LDL but also from triglyceride-rich remnant particles and endothelial cells. The antioxidant effects of HDL are now well established and have been demonstrated in a number of experimental systems (Kontush and Chapman, 2010).

## HDL antiglycative activity

We have also shown that HDL can impede the modification of LDL by glycation *in vitro*, and that this anti-glycative function of HDL is more marked with HDL obtained from people with higher serum PON1 activity (Younis et al., 2013). We noted in these experiments that LDL is relatively resistant to glycation in the absence of oxygen, such that supraphysiological glucose concentrations are required. Oxidation appears to accompany *in vitro* glycation and the process is best regarded as glycoxidation. The lipid peroxidation of LDL that accompanies *in vitro* glycation is also impeded in the presence of HDL. Adduction of lipid peroxidation products to the ε amino groups of apoB lysine residues *in vivo* may render these groups more susceptible to combination with glucose. Thus, *in vivo* exposure of LDL to oxygen free radicals may predispose to glycation and explain the observed high levels of circulating glycated LDL. The effect of HDL on glycation may thus be related to its anti-oxidative function. An alternative hypothesis is that the oxidized analog of glucose, gluconolactone, is more involved in apoB glycation, and that this step might be affected by PON1's lactonase activity.

## Paraoxonase 1

PON1 is produced in the liver and circulates on HDL. There is a significant body of evidence to support a role for PON1 in atherosclerosis, and in particular against oxidation, not least its capacity to hydrolyze lipid hydroperoxides.

We demonstrated that the HDL fraction containing PON1 was most active in impeding Cu^2+^ induced lipid peroxide accumulation on LDL (Mackness et al., 1993). It has since been suggested that it is not PON1 which is responsible for this effect, an argument supported by reports that more highly purified PON1 isolated from HDL and recombinant water-soluble variants of PON1 do not hydrolyze lipid peroxides (Draganov et al., 2005; Kontush and Chapman, 2010). It is however exceptionally difficult to separate PON1 from other HDL components, such as apolipoprotein AI (apoAI) and phospholipase A2 (PLA2), without subjecting it to conditions which might affect its catalytic activity (Ben-David et al., 2015). Similarly, the increased polarity of recombinant PON1 would be expected to compromise its ability to hydrolyze hydrophobic substrates (Harel et al., 2004; Draganov et al., 2005; Bajaj et al., 2014). More lipophilic recombinant PON1 might be expected to have improved functionality, but is more difficult to isolate, a factor which will prove important in the development of recombinant PON1 for therapeutic use (Bajaj et al., 2014). Interestingly, HDL from avian species, which lacks paraoxonase activity, does not protect human LDL against lipid peroxidation (Mackness et al., 1998). Similarly, *PON1* knockout mice are more susceptible to atherosclerosis and their HDL is less able to prevent the accumulation of lipid peroxides on human LDL (Shih et al., 1998), whereas transgenic rodent models expressing human *PON1* are protected against atherosclerosis (Tward et al., 2002; Zhang et al., 2010).

Epidemiological studies have consistently shown that PON1 activity is independently inversely associated with coronary events (Mackness et al., 2003; Wang et al., 2012). A recent meta-analysis, which considered 47 such studies, reported that PON1 activity was 19% lower in patients suffering from coronary heart disease than in unaffected controls (Wang et al., 2012). Other prospective studies expanded on the negative correlation between PON1 activity and coronary heart disease by also reporting circulating levels of lipid peroxidation products, linking these to PON1 anti-oxidative activity (Bhattacharyya et al., 2008; Karlsson et al., 2015).

A number of medical conditions including diabetes mellitus, chronic kidney disease, familial hypercholesterolaemia and inflammatory arthritides are associated with both decreased serum PON1 activity and increased CVD risk (Soran et al., 2009). PON1 activity is decreased in both type 1 and 2 diabetes (Mackness et al., 2000, 2002) and lower levels are associated with microvascular complications (Mackness et al., 2000, 2002; Hofer et al., 2006).

PON1 has several genetic polymorphisms, the most extensively researched of which is the R192Q variant. This polymorphism has a substantial effect on PON1's capacity to hydrolyze paraoxon and homozygotes and heterozygotes for the R allele are more resistant to parathion (paraoxon is formed from this widely used organophosphate pesticide once it enters the body) than QQ homozygotes (Mackness et al., 2001; Cherry et al., 2002; Wheeler et al., 2004). Other activities of PON1, such as phenyl acetate hydrolysis, which proceed at faster rates, are, however, unaffected by the R192Q polymorphism. In the case of the protective effect of HDL against LDL oxidation, HDL from 192QQ homozygotes is most effective in preventing the accumulation of lipid peroxides on LDL (Mackness et al., 1997; Durrington et al., 2001), but this effect is small in comparison to the huge variation in serum PON1 activity. Nonetheless 192QQ homozygotes have been reported to have reduced CVD risk (Mackness et al., 2001; Wheeler et al., 2004). This inverse association is, however, within the range which could be explained by publication bias, but it does not deny that the wider range of PON1 activities encountered in populations resulting from other genetic and acquired influences are relevant to the development of atherosclerosis. It certainly indicates that the substrate specificity involved in the antiatherogenic effect of PON1 is not greatly influenced by the R192Q polymorphism.

*PON1* and *PON2* genotype have been linked with susceptibility to develop diabetes (Rozenberg et al., 2008), glycaemic control (Hegele et al., 1997), and diabetic microvascular complications (Mackness et al., 2000; Hofer et al., 2006; Wang et al., 2013). It has been suggested that this association reflects a role for oxidation in pancreatic β cell dysfunction or microvascular disease. Alternatively, it might reflect an ability of HDL / PON1 to prevent post-translational protein glycation.

Parenteral administration of partially purified PON1 can ameliorate experimental atherosclerosis. Recombinant PON1 might also be used for this purpose, if its properties can be retained during isolation (Draganov et al., 2005; Bajaj et al., 2014). Intraperitoneal injection of recombinant PON1 in mice increased cholesterol efflux capacity and HDL aryl esterase and lactonase activities, and decreased macrophage mediated LDL oxidation (Rosenblat et al., 2011).

## Paraoxonase 2 and paraoxonase 3

PON2 is almost exclusively found intracellularly, whereas PON3 is also associated with HDL, albeit in lesser quantities than PON1. The primary hydrolytic activity of PON3 is also as a lactonase (Draganov et al., 2005). *PON3* knockout mice are also more susceptible to atherosclerosis (Zhang et al., 2010), but the reason for its evolutionary conservation is currently unclear.

## Apolipoprotein AI

ApoAI is essential both for the structure of HDL and the maintenance of the lipid environment in which enzymes such as PON1 and lecithin: cholesterol acyl transferase (LCAT) can operate (Rye and Barter, 2014). It is therefore likely to have a major role in the antioxidant effects of HDL. ApoAI plays a central role in the redox inactivation of lipid hydroperoxides which follows their transfer to HDL. ApoAI also creates a safe environment for the release of lysophospholipids and their subsequent transfer to the liver. Despite experimental evidence that lipid-protein complexes containing only apoAI can protect LDL against oxidation (Karlsson et al., 2015), neither animal models nor human genetic disorders have provided convincing evidence that apoAI's anti-atherogenic effects are independent of changes in HDL levels (Duverger et al., 1996; Plump et al., 1997). Interestingly, apoAI-mimetic peptides create circulating lipid complexes, which associate with other components of HDL such as PON1 (Mishra et al., 2013). This may provide a means of enhancing circulating PON1 activity.

## Apolipoprotein AII (apoAII)

Apolipoprotein AII (apoAII)-containing HDL particles tend to be larger and possess less antioxidant activity than those with higher apoAI content (Karlsson et al., 2015). There is evidence from both animal models and human studies to suggest that apoAII might actually suppress PON1 binding to HDL (Litvinov et al., 2012). Mice expressing human apoAII and apoAI are more susceptible to atherosclerosis than those expressing apoAI alone.

## Other apolipoproteins

Other apolipoproteins associated with HDL may act alongside apoAI to inhibit lipid hydroperoxide accumulation. Apolipoprotein E (apoE) appears to display this anti-oxidative activity (Miyata and Smith, 1996), while apolipoprotein M (apoM) has recently been reported to display anti-oxidative functionality in transgenic mice in addition to facilitating PON1 activity (Elsøe et al., 2012; Borup et al., 2015). There is currently little evidence that apolipoprotein J (apoJ) contributes to the anti-oxidative activities of HDL, but it does appear to possess a variety of functions, including endothelial protection.

## Myeloperoxidase (MPO)

Cellular systems contributing to oxidative stress *in vivo* include MPO, NADPH oxidase, nitric oxide synthase, and lipoxygenase (Karlsson et al., 2015). It has recently been proposed that MPO and PON1 form a ternary complex with HDL, where the opposing activities of MPO and PON1 determine its oxidation state and whether HDL is pro- or anti-inflammatory/atherogenic (Huang et al., 2013). The ratio between these enzyme activities has also been proposed as a marker of HDL functionality and to predict coronary risk (Haraguchi et al., 2014).

## Glutathione peroxidase (GSPx)

Although, its levels do not appear to affect coronary heart disease risk, GSPx is found associated with HDL, where it has the ability to reduce lipid hydroperoxides (Karlsson et al., 2015). Similarly, trypanosome lytic factor present in higher density HDL also exhibits peroxidase activity and may contribute to the anti-oxidative properties of HDL (Karlsson et al., 2015).

## Phospholipase A2

Most PLA2 is associated with LDL, where its activity is an independent risk factor for coronary heart disease (Rosenson and Hurt-Camejo, 2012). There is however, no evidence that the minor fraction of PLA2 activity on HDL is pro-atherogenic. Furthermore, PLA2 has overlapping activity with PON1 and it remains unclear just how much of the hydrolysis of platelet activating factor by HDL is due to PLA2 and how much to PON1 (Rodrigo et al., 2001). PLA2 on HDL is likely to contribute anti-oxidative activity by the same mechanism as PON1, by hydrolyzing lipid hydroperoxides. This activity, which would be pro-atherogenic on LDL in the presence of apoB, may be antiatherogenic in the environment provided by HDL.

## Lecithin: Cholesterol Acyl transferase

Similarly, there is currently limited evidence to support a role for LCAT in the antioxidative activity of HDL (Holleboom et al., 2012), but its association with HDL and, like PON1 and PLA2, its role in generating lysophospholipids, does contribute to the hypothesis that HDL provides a safe place to release lysolipids otherwise potentially damaging to cell membranes and other lipoproteins.

## Cholesteryl ester transfer protein (CETP) and phospholipid transfer protein (PLTP)

The antioxidant activity of HDL occurs following the transfer of lipid hydroperoxides to HDL. Experimentally no additional facilitator of transfer to HDL is required, but some CETP and PLTP are likely to remain in physical association with HDL after its isolation. CETP can accelerate the transfer of both cholesteryl ester and phospholipid hydroperoxides (Christison et al., 1995). CETP and/or PLTP may thus be important for the anti-oxidative effect of HDL *in vivo*. Like PON1, PLTP is found predominantly in small, dense HDL, where it is able to interact with apolipoproteins implicated in anti-oxidative function, including apoAI, apoAII, and apoJ (Karlsson et al., 2015).

## Conclusion

The capacity of HDL to protect LDL against oxidative modification is considerable, but its potential therapeutic use to prevent atherosclerosis is as yet unfulfilled. The interaction of lipids with apoAI in HDL provides a lipoprotein particle capable of acquiring potentially toxic lipids and holding them in an environment where they may be safely hydrolyzed and from which they may be released to the liver for elimination. PON1, PLA2, and LCAT are present at higher concentrations in small, dense, protein-rich HDL (Karlsson et al., 2015), and HDL particles are therefore heterogeneous in their anti-oxidative capacity. PON1 is likely to be critical to the antioxidative capacity of HDL, but is likely to require a lipid environment to support its activity. Separation of HDL from PON1 disrupts this and the necessary environment is only imperfectly present with the currently available water-soluble recombinant forms of PON1. Acting together on HDL, PON1, apoAI, apoM, and PLA2 in conjunction with CETP and other lipid transfer proteins probably create a system with both antioxidative and antiglycative properties (see Figure 1).

### Conflict of interest statement

The authors declare that the research was conducted in the absence of any commercial or financial relationships that could be construed as a potential conflict of interest.
